# Collapsing a Perfect Superposition to a Chosen Quantum State without Measurement

**DOI:** 10.1371/journal.pone.0103612

**Published:** 2014-08-01

**Authors:** Ahmed Younes, Mahmoud Abdel-Aty

**Affiliations:** 1 Department of Mathematics and Computer Science, Faculty of Science, Alexandria University, Alexandria, Egypt; 2 University of Science and Technology, Zewail City of Science and Technology, Cairo, Egypt; 3 Department of Mathematics, Faculty of Science, Sohag University, Sohag, Egypt; Washington State University, United States of America

## Abstract

Given a perfect superposition of 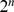 states on a quantum system of 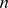 qubits. We propose a fast quantum algorithm for collapsing the perfect superposition to a chosen quantum state 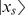 without applying any measurements. The basic idea is to use a phase destruction mechanism. Two operators are used, the first operator applies a phase shift and a temporary entanglement to mark 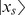 in the superposition, and the second operator applies selective phase shifts on the states in the superposition according to their Hamming distance with 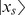. The generated state can be used as an excellent input state for testing quantum memories and linear optics quantum computers. We make no assumptions about the used operators and applied quantum gates, but our result implies that for this purpose the number of qubits in the quantum register offers no advantage, in principle, over the obvious measurement-based feedback protocol.

## Introduction

Generation of non-classical states of light compatible with atomic quantum memory has been an outstanding challenge driven by various applications in quantum information processing [1]. Various approaches to generation of single photon states compatible with atoms have been pursued [2]: single atoms in free space [3] and in high-finesse cavities [4] and atomic ensembles [5], and non-classical features such as photon antibunching and violation of classical inequalities have been demonstrated.

On the other hand, several specific quantum algorithms have been discovered (see [6] and references therein), providing “quantum speedup” with respect to their fastest classical counterparts. A quantum analog of the computational complexity theory has been developed [7]–[8], with the introduction of complexity classes of easy and hard problems, the notion of difficulty being now with respect to the number of required operations on a quantum, instead of classical, computer. A new formulation of monotonically convergent algorithms which allows to optimize both the control duration and the field influence has been presented [9]. They apply this algorithm to the control of spin systems in Nuclear Magnetic Resonance and show how to implement CNOT gates in systems of two and four coupled spins. Also, a new formulation of quantum algorithm which allows to distribute amplitudes over two copies of small quantum subsystems has been proposed [10], where a standard algorithm designs a new method of a fixed number of copies and applied to the control of multi-qubit system.

The problem of how to perform quantum operations on a perfect superposition state containing a multi-qubit plays a fundamental role in obtaining a certain quantum state without applying any measurements. Our approach for detecting quantum state is based on the possibility of applying the phase shifts operators which based on Hamming Distance. Here we provide new tools for the building-up of unitary transformations from simple gates. To do that, we consider a given quantum system 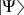 of 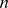 qubits in a perfect superposition, 
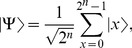
(1)such that 
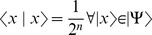
, i.e. applying measurement on 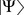 gives any 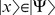 with equal probability 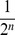. It is required to make 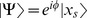, where 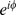 is some global phase shift, for a certain chosen 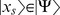 such that 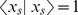, i.e. the probability of 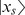 is certainty instead of 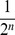 without applying any measurements.

We begin in Sec. 2, by showing that it is possible to obtain an improvement for the simple case by presenting the elementary operations, i.e. the basic gates used in the proposed algorithm, gates acting on a the qubits. In Sec. 3 we show that, if we allow the application of some phase operators on a superposition of multi-qubit state, rather than an incoherent mixture, it is possible to obtain a perfect quantum state. Then, in Sec. 4, we show that there are deep connections between the proposed algorithm and quantum unsolved problems for post-processing and we conclude in Sec. 5. See Table 1 for a list of symbols and their definitions.

**Table 1:** List of symbols and their definitions.

Symbol	Definition
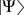	quantum system of 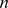 qubits
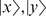	quantum states such that 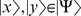
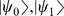	quantum sub-systems such that 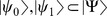
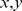	bitwise representation of 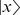 and 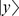
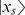	chosen quantum state such that 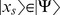
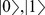	a single qubit state
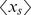	binary representation of 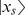
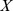	a single qubit negation gate
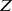	a single qubit phase shift gate
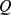	a single qubit square root of NOT with global phase shift gate
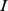	a single qubit Identity gate
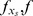	a Boolean function that evaluates to 1 for 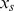
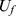	an operator that marks quantum states by entanglement according to 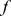
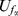	an operator that marks quantum states by phase shift according to 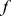
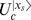	an operator that applies specific phase shifts according to 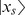
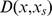	Hamming distance between 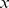 and 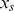

## Discussion

In this section, the basic gates used in the proposed algorithm will be defined [11]. Some gates are acting on a single qubit of the system. Some gates are acting on the 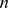 qubit register and other gates are acting on the 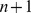 qubit register.

Three gates acting on single qubits will be used, negation gate 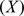, phase shift gate 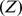, and a square root of not with a global phase shift gate 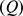. The first quantum gate 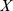 that performs similarly to the classical 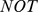 gate, i.e. it inverts 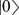 to 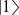 and 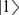 to 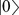. The phase shift operator 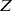 is used to apply a phase shift of -1 on the amplitude of the state 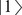 and leaves the amplitude of 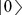 with no change.

Such operation will be used to apply a phase shift of 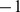 on a subspace of the system entangled with state 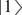 as follows, 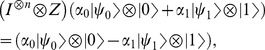
(2)where 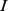 is the identity operator, 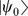 and 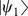 are sub-systems entangled with 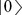 and 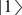 respectively.

The 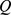 gate is a quantum gate that performs a square root of not with a global phase shift. Applying the 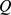 gate on a qubit in state 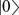 or 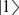 will produce a qubit in a perfect superposition with some phase shift. Applying 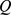 gate twice produces the negation of the original input with some global phase shift. The effect of applying 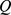 gate on a single qubit can be understood as follows, 
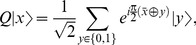
(3)where 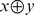 is the bitwise-XOR of 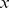 and 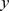, and 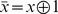. Applying 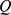 twice gives the following, 
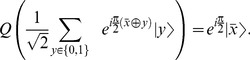
(4)


In general, the effect of applying 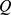 gate on 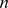-qubit quantum register can be understood as follows, 
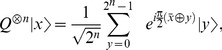
(5)where 
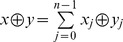
 is the summation of the bitwise-XOR of 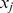 and 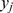. Applying 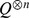 twice gives, 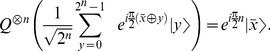
(6)


In the literature, there are two ways used to mark certain states in a superposition. One way is to conditionally apply certain phase shifts on the marked states [12] and the other way is to entangle the required states with certain state of an extra working qubit [13]. An operator 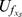 is used in both cases to recognize the state(s) to be marked, where 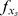 is a Boolean function evaluates according to the following, 
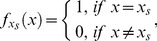
(7)


For short, 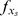 will be written as 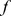 in the following sections. To mark a state using a phase shift of 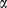, an operator 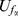 of the following effect has been used, 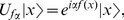
(8)and to mark a state by entanglement, an operator 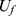 of the following effect has been used, 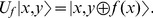
(9)


In the proposed algorithm, a combination of both methods will be used where an operator of the form 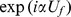 is used, where 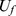 has the following effect, 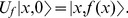
(10)


Using Taylor's expansion, 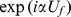 can be re-written as [14], 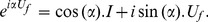
(11)


The effect of applying the operator 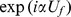 on a superposition of 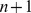 qubit register can be understood as follows, 
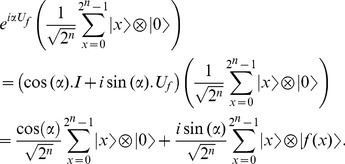
(12)


The operator 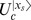 is an operator used to apply specific phase shifts on the states included in the superposition based on the Hamming distance between these states and a given state 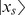. The operator 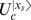 applies phase shifts according to the following rule, 
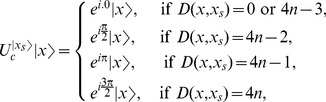
(13)where 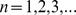 and 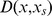 is the Hamming distance between 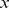 and 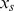 where 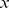 and 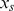 are vectors of length 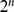.

To construct 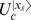, for a given 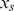, choose the corresponding row/column for that state from Table 2 and insert these values as the diagonal in a zero elements matrix. For example, if 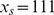, then the corresponding matrix is, 
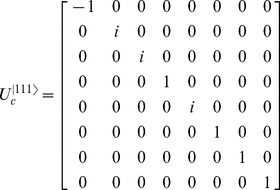
(14)


**Table 2:** Table of phase shifts based on Hamming Distance for 3-qubit states.

	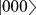	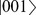	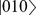	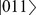	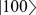	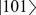	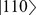	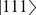
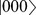	1	1	1	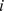	1	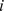	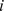	−1
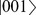	1	1	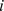	1	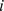	1	−1	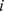
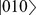	1	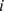	1	1	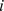	−1	1	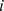
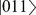	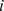	1	1	1	−1	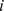	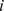	1
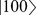	1	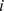	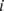	−1	1	1	1	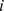
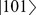	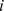	1	−1	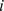	1	1	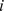	1
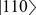	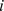	−1	1	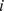	1	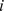	1	1
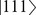	−1	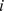	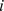	1	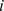	1	1	1

To simplify the construction of 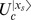, instead of choosing the appropriate row/column from Table 2. The same construction can be done as follows, 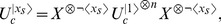
(15)where 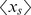 is the bit representation of 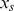, and 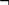 is the bitwise negation operator. For example, if 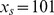, then, 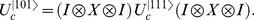
(16)


## Method and Algorithm

Given a superposition of 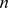 qubits and a state 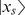. It is required to make the superposition collapse to 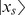 without applying any measurement. The operations of the algorithm is applied as follows,
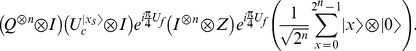
(17)


Let 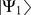 be a quantum register of 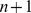 qubits, where the first 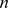 qubits are in a superposition and the last qubit is initialized to state 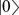, 
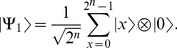
(18)


The steps of the algorithm are as follows:

- 1-Apply 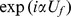 taking 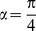. 
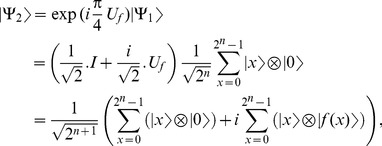
(19)where 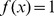 if 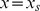 and 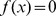 otherwise. Then the system can be re-written as follows, 
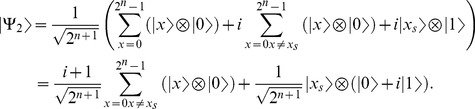
(20)
- 2- Apply 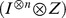. 
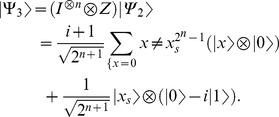
(21)
- 3- Apply 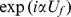 taking 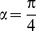. 
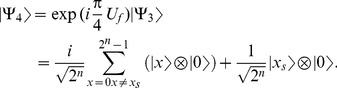
(22)
- 4- Apply 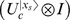. 
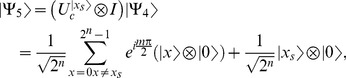
(23)where 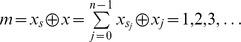. The system can be re-written as, 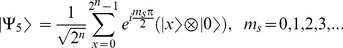
(24)
- 5- Apply 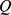 gate on each of the first 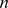 qubits as shown in Eqn. 6.
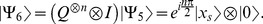
(25)

## Results

The above algorithm can be used in quantum storage protocols based on photon echo techniques which rely on the reversible absorption of a single photon pulse in an inhomogeneously broadened media [15]. After absorption, the single photon state is mapped onto a single collective atomic excitation at the optical transition,
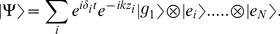
(26)


In Eq. (26), we denote by 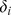 the detuning of atom i with respect to the central frequency of the photon and 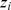 the position of atom 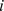. This collective state rapidly dephases, since each term acquires a phase 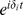. The goal of the quantum memory protocols is to engineer the atomic system such that this inhomogeneous dephasing can be reversed. If this rephasing can be implemented, the light is re-emitted in a well defined spatio-temporal mode when the atoms are all in phase again, as a result of a collective interference between all the emitters. The rephasing of the dipoles can be triggered by optical pulses, as it is the case in traditional photon echo techniques. These techniques, while very successful to store classical light [16] and as a tool for high resolution spectroscopy [17], suffer from strong limitations for the storage of single photons.

## Conclusion

We want to end with a summary and a discussion of a number of open questions related to the proposed algorithm and its possible applications to problems beyond the quantum memory. The underlying idea of the proposed algorithm is very general and consists of employing Hamming distance to transform the superposition state into a specific quantum state. The crucial advantage is that any required quantum state can now be exactly created using these simple operations. Novel type of applications can be formed in quantum storage protocols based on photon echo techniques. Also, because the dynamical model used here can be equally realized in multi-qubit models, an exponential propagation of quantum excitation along a large number of qubits is in principle possible.
